# 1D Mathematical Modelling of Non-Stationary Ion Transfer in the Diffusion Layer Adjacent to an Ion-Exchange Membrane in Galvanostatic Mode

**DOI:** 10.3390/membranes8030084

**Published:** 2018-09-19

**Authors:** Aminat Uzdenova, Anna Kovalenko, Makhamet Urtenov, Victor Nikonenko

**Affiliations:** 1 Department of Computer Science and Computational Mathematics, Karachaevo-Cherkessky State University Named after U.D. Aliev, Karachaevsk 369202, Russia; uzd_am@mail.ru; 2 Department of Applied Mathematics, Kuban State University, Krasnodar 350040, Russia; savanna-05@mail.ru (A.K.); urtenovmax@mail.ru (M.U.); 3 Department of Physical Chemistry, Kuban State University, Krasnodar 350040, Russia

**Keywords:** ion-exchange membrane, galvanostatic mode, mathematical modelling, Nernst–Planck and Poisson equations, transition time, electroconvection

## Abstract

The use of the Nernst–Planck and Poisson (NPP) equations allows computation of the space charge density near solution/electrode or solution/ion-exchange membrane interface. This is important in modelling ion transfer, especially when taking into account electroconvective transport. The most solutions in literature use the condition setting a potential difference in the system (potentiostatic or potentiodynamic mode). However, very often in practice and experiment (such as chronopotentiometry and voltammetry), the galvanostatic/galvanodynamic mode is applied. In this study, a depleted stagnant diffusion layer adjacent to an ion-exchange membrane is considered. In this article, a new boundary condition is proposed, which sets a total current density, *i*, via an equation expressing the potential gradient as an explicit function of *i*. The numerical solution of the problem is compared with an approximate solution, which is obtained by a combination of numerical solution in one part of the diffusion layer (including the electroneutral region and the extended space charge region, zone (I) with an analytical solution in the other part (the quasi-equilibrium electric double layer (EDL), zone (II). It is shown that this approach (called the “zonal” model) allows reducing the computational complexity of the problem tens of times without significant loss of accuracy. An additional simplification is introduced by neglecting the thickness of the quasi-equilibrium EDL in comparison to the diffusion layer thickness (the “simplified” model). For the first time, the distributions of concentrations, space charge density and current density along the distance to an ion-exchange membrane surface are computed as functions of time in galvanostatic mode. The calculation of the transition time, *τ*, for an ion-exchange membrane agree with an experiment from literature. It is suggested that rapid changes of space charge density, and current density with time and distance, could lead to lateral electroosmotic flows delaying depletion of near-surface solution and increasing *τ*.

## 1. Introduction

Mathematical modelling based on the Nernst-Planck and Poisson (NPP) equations built in one-, two- and three-dimensional geometry is largely used for describing ion transport in electrode and membrane systems. The boundary-value problems involving these equations are mathematically complicated, since a small parameter is present in the Poisson equation (in its dimensionless form) [1]. The most models are developed for the potentiostatic mode, where electric regime is set through the potential difference between two equipotential planes enveloping an electrode/membrane and parallel to it [2,3,4,5,6,7,8,9,10,11,12,13,14,15,16,17]. The relationship between the local electric potential and ion concentrations is given by the Poisson equation. Though in the practice of electrochemical characterization of membranes and electrodes (chronopotentiometry, voltammetry, impedancemetry) the galvanostatic or galvanodynamic mode is frequently used [18,19,20]. However, ion transfer modelling in this mode encounters serious difficulties associated with the method of specifying the current strength, as a parameter determining the electrical regime.

For this reason, the existing mathematical models for the galvanostatic mode are based either on the inverse problem method or on the simplifying assumption of local electroneutrality (LEN) [21,22,23,24,25,26]. The implementation of the inverse problem method requires multiple solutions of the equation system under the potentiostatic condition for a given value of the current density, which requires large computational costs.

In References [24,25], to describe the galvanostatic mode, the decomposition of two-dimensional NPP equations was performed on the basis of the LEN assumption. 2D modelling of ion and water transfer in the galvanostatic mode requires the setting of an integral boundary condition for the electric current density—as the integral of the local normal current density, *i*_n_, which in general case varies with the longitudinal coordinate. The solution of the corresponding mathematical problem in the case where *i*_n_ ≠ const is possible, when using the current stream function method [24,25]. The application of this method is based on a strict mathematical analogy between the electric current flow and the fluid flow, while considering that stream functions are widely used in fluid mechanics.

However, the approaches based on the LEN assumption do not allow taking explicitly into account the effect of the space charge region (SCR), which is formed at the solution/membrane boundary. The agreement between the simulation and experiment results is achieved by introducing into the mathematical description some empirical parameters and additional assumptions [23,26].

Manzanares et al. [27] studied the change in the structure of the nonequilibrium electric double layer (EDL) at the ion-exchange membrane/solution interface under the action of an applied electric current. The study was based on the numerical solution of the NPP equations with a boundary condition, which allowed setting the total current density as the parameter governing the electric regime in the system. Unlike potentiostatic (potentiodynamic) models [2,3,4,5,6,7,8,9,10,11,12,13,14,15,16,17], where the potential difference was set, in Reference [27] the time derivative of the electric potential gradient at the outer edge of the diffusion layer was specified as an explicit function of the current density.

In this paper, we propose a 1D model similar to that developed in Reference [27]. Our model is also based on the NPP equations for the galvanostatic mode, i.e., the total electric current is the governing parameter. However, instead of the time derivative of the electric potential gradient at the edge of depleted diffusion layer, which is specified in Reference [27], we specify at this boundary the electric potential gradient expressed as an explicit function of the total current density. The resulting formulation of the boundary condition is simpler, since it does not require integration over time. This simplifies the numerical solution and allows a rather easy application of the Comsol Multiphysics package not requiring an additional interface for the time integral calculation. Moreover, our boundary condition involves the ion transport number in the membrane, thus quantifying possible non-ideal membrane permselectivity. The boundary condition proposed in Reference [27] requires the calculation of the counterion and co-ion concentrations and their gradients, as well as the potential gradient in the membrane.

Another novelty of our paper is a simplification of the solution of the boundary-value problem, where a numerical solution in one part of the system (comprising the electroneutral zone and the extended SCR) is combined with an approximate analytical solution in the other part (in the equilibrium zone of the EDL). Rubinstein and Zaltzman [3] used a similar approach, however they found a numerical solution only in the electroneutral zone, while an approximate analytical solution was found for the overall SCR and then applied as a boundary condition for the numerical solution. This approach allowed Rubinstein and Zaltzman to obtain a large number of physicochemical results concerning the mechanisms of mass transfer in membrane systems in potentiostatic mode [3,28,29]. Their results, in turn, stimulated other authors to develop productive models giving new insights into the theory of mass transfer in ion-exchange membrane and nano/microfluidic systems [4,5,6,7,8,9,10,11,12,13,14]. 

The importance of simplifying the solutions for the ion and water transfer problems, which take into account the effect of SCR, is related to the fact that these problems, both galvanostatic and potentiostatic, are computationally very complex. The quasi-equilibrium EDL of a thickness *λ* scaled by the Debye length is characterized by huge gradients of ion concentrations and electric potential. The computational complexity of the problem, as well as computational costs increases rapidly when the ratio of *λ* to the diffusion layer thickness *δ* decreases [8]. The later occurs with increasing the electrolyte concentration, since *λ* decreases. For this reason, the most computations in literature were carried out for the *λ*/*δ* values, which are far from the parameters of real membrane systems (at least in electrodialysis applications).

## 2. Mathematical Models

**Figure 1:**
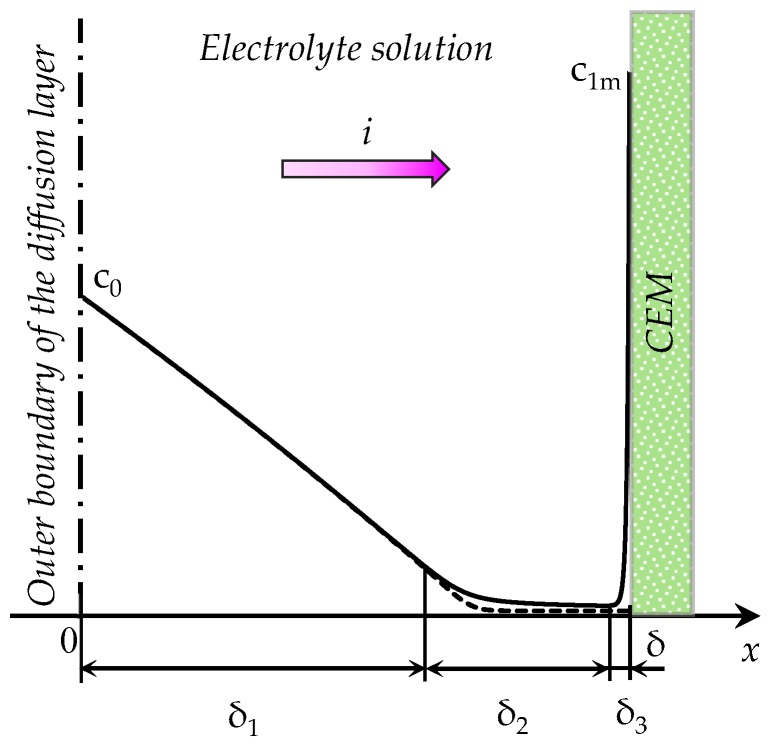
Schematic concentration profiles of cations (*c*_1_, the solid line) and anions (*c*_2_, the dashed line) in the diffusion layer adjacent to the surface of a cation-exchange membrane (CEM) [2]. The current density *i* is flowing across the system; the electrolyte concentration in the bulk solution, *c*_0_; the cation concentration at the solution/CEM boundary, *c*_1m_; the minimum concentration of cations, *c*_1s_; *δ*_1_, *δ*_2_ and *δ*_3_ are the thicknesses of different diffusion layer regions: The electroneutral region, the extended SCR and the quasi-equilibrium electric double layer (EDL), respectively.

The system under consideration involves a diffusion boundary layer of a binary electrolyte solution at the surface of a cation-exchange membrane (CEM). Let *x* be the coordinate normal to the membrane surface, varying from 0 (the solution bulk = outer boundary of the diffusion layer) to *δ* (the solution/membrane boundary), Figure 1. A constant value of the current density, *i = const*, is set at the left- (*x* = 0) or right-hand (*x* = 1) boundary.

The following three regions can be distinguished in the structure of the diffusion layer [2,30]: The electroneutral region, the extended SCR and the quasi-equilibrium EDL. In Figure 1 the thicknesses of these regions are denoted by *δ*_1_, *δ*_2_ and *δ*_3_, respectively. The scheme in Figure 1 is based on numerical calculations of the steady-state ion concentration distribution in the diffusion layer at the cation-exchange membrane according to the (potentiostatic) model proposed by Rubinstein and Shtilman [2]. According to Reference [2] and other studies [3,4,5,6,7,8,9,10,11,12,13,14], the concentration of anions *c*_2_ decreases monotonically when approaching the membrane to reach a relatively low value within the membrane. This low value is caused by the Donnan co-ion exclusion (the electrostatic repulsion of the ions, whose charge has the same sign as that of the fixed ions). The concentration of cations *c*_1_ decreases first being very close to the co-ion concentration (in the electroneutral region), passes through a minimum, and then sharply increases to reach a value *c*_1m_ at the point *x* = 1. The value of *c*_1m_ is close to the concentration of the fixed ions in the membrane [2], which is of the order of 1 mol/L (=10^3^ mol/m^3^). The sharp increase of *c*_1_ in the vicinity of the membrane (in the quasi-equilibrium part of the EDL) is due to the condition of continuity of concentrations when passing across the solution/membrane boundary [2,3,4,5,6,7,8,9,10,11,12,13,14].

In this study, we consider three variants of mathematical description of 1D non-stationary ion transfer in the diffusion layer (Figure 1) in the galvanostatic mode:

(1) The “primary” model, which differs from potentiostatic models by the boundary condition: The potential gradient determined by the current density is given instead of the potential drop. Only a numerical solution is obtained for this model.
(2) The “zonal” numerical-analytical model, in which the diffusion layer is split into two zones, where the solution is determined separately. The first zone includes the electroneutral region *δ*_1_ and the extended SCR *δ*_2_, the second zone is the equilibrium part of EDL *δ*_3_ (Figure 1). The model considers the change in the thickness of both zones with time, when concentration polarization develops under an applied current density *i*.
(3) The “simplified” model, in which the thickness of the equilibrium part of EDL is assumed equal to zero: *δ*_3_ = 0. Since the sum of the thicknesses of all zones is given (equal to *δ*), in this simplification the thickness of the first zone is overestimated. It is shown, that at relatively large values of the inlet concentration of the electrolyte solution, this overestimation can be neglected, and the calculations are considerably simplified.

### 2.1. The “Primary” Galvanostatic Model

#### 2.1.1. The System of Equations and the Boundary Conditions

The mathematical model of the non-stationary ion transfer includes the Nernst-Planck equation, Equation (1); the matter conservation equation (the continuity equation), Equation (2); the Poisson equation, Equation (3); and the equation for the total electric current density, including the displacement current [27,31], Equation (4). Convective transfer is not considered. This equation system for a binary electrolyte in a dimensionless form reads as follows:

(1) $$j_{i}=-z_{i}D_{i}c_{i}\frac{\partial \varphi }{\partial x}-D_{i}\frac{\partial c_{i}}{\partial x},\;i=1,\;2,$$

(2) $$\frac{\partial c_{i}}{\partial t}=-\frac{\partial j_{i}}{\partial x},\;i=1,\;2,$$

(3) $$\varepsilon \frac{\partial^{2}\varphi }{\partial x^{2}}=-\left(z_{1}c_{1}+z_{2}c_{2}\right),$$

(4) $$i=\left(z_{1}j_{1}+z_{2}j_{2}\right)-\varepsilon \frac{\partial^{2}\varphi }{\partial x\partial t},$$

where the dimensionless spatial coordinate, *x*, is normalized to the thickness of the diffusion layer *δ*; the time, *t*, to the characteristic time of electrolyte diffusion through a layer of thickness *δ*, *δ*^2^/*D*; the molar concentration of *i*-th ion, $c_{i}$, to the electrolyte concentration in the bulk solution, $c_{0}$; the electric potential φ, to the value RT/F; the current density, i, to the value *Dc*_0_*F*/*δ*; the ion flux, *j_i_*, to the value *Dc*_0_/*δ*; the individual ion diffusion coefficients, *D_i_*, to the electrolyte diffusion coefficient, *D = D*_1_*D*_2_*(z*_1_ *− z*_2_*)/(D*_1_*z*_1_ *− D*_2_*z*_2_*)*. The system of equations contains a small dimensionless parameter $\varepsilon =\varepsilon_{r}\varepsilon_{0}RT/\left(c_{0}\delta^{2}F^{2}\right)$ at the derivative in the Poisson equation. This parameter can be presented as the *ε =* 2(*L_D_/δ*)^2^ ratio, where $L_{D}=\sqrt{RT\varepsilon_{0}\varepsilon_{r}/\left(2c_{0}z_{1}^{2}F^{2}\right)}$ is the Debye length. Here, *z_i_* is the charge number of the *i*-th ion; *ε_0_* is the dielectric permittivity of vacuum; *ε_r_* is the solution relative permittivity (assumed constant); *F* is the Faraday constant; *R* is the gas constant; *T* is the absolute temperature. In Equations (1) and (2) φ, *c*_1_ and *c*_2_ are unknown functions of *t* and *x*.

In the case of laminar flow of the solution between two parallel smooth membranes of length *L*, the average (effective) value of *δ* can be estimated using the Leveque equation *δ* = (*H*/1.47)(*LD*/(*H*^2^*V*_0_))^1/3^ [1,32], where *H* is the channel width (the intermembrane distance); *V*_0_ is the average flow velocity of the solution between the membranes. This value determines the average limiting current density over the length *L* in conditions where the diffusion layer thickness varies along the channel length [32].

In Equation (4), the term $i_{c}=(z_{1}j_{1}+z_{2}j_{2})$ is the conduction current density, while $i_{d}=-\varepsilon \frac{\partial^{2}\varphi }{\partial x\partial t}$ is the displacement current, which is associated with time formation of a space charge—as it follows from the Poisson equation, $\frac{\partial \rho }{\partial t}=-\varepsilon \frac{\partial^{3}\varphi }{\partial x^{2}\partial t}$. As far as *ρ* changes with time, the conduction current density varies as a function of distance, according to the charge continuity equation [33]:

(5) $$\frac{\partial \rho }{\partial t}+\frac{\partial i_{c}}{\partial x}=0.$$

However, the total current density *i* is not a function of *x* [27].

As it was mentioned above, the current density condition may be set at the left- or right-hand boundary. Let us first consider the case where this condition is set at *x* = 1.

At *x =* 0 (the outer edge of the diffusion layer), the following conditions are applied:

(6) $$c_{i}(0,t)=1,\;i=1,\;2,$$

(7) φ(0,t)=0.

At the right-hand boundary, *x =* 1 (the solution/membrane interface), two parameters are specified. One of them is the counterion concentration *c*_1m_, which is set as a constant value *N*_c_ times greater than the bulk solution concentration:

(8) $$c_{1}(1,t)=c_{1m}=N_{c}.$$

Condition (8) was first proposed by Rubinstein and Shtilman [2]. Together with Equations (1–3) and Conditions (6–8), the authors of Reference [2] have used condition $\varphi (1)=\varphi_{1}$ for solving the boundary-value problem in steady state. The problem relates to the singularly perturbed type of differential equations [34], due to the small coefficient in the Poisson equation.

In our formulation of the problem, the current density *i* is used as the parameter determining the electric regime in the system. This parameter is entered via the potential gradient ∂φ/∂x, which is specified at the solution/membrane boundary (*x =* 1) as an explicit function of *i*, for solution of the Poisson equation. Two variants of setting this function are used. Both functions are mathematically equivalent, but their use leads to numerical solutions, which differ in accuracy (Section 2.1.4).

The first variant applies Equation (9):

(9) $$\left(\frac{\partial \varphi }{\partial x}\right)\left(1,t\right)=-\left[\frac{1}{z_{1}c_{1}}\left(\left(i+\varepsilon \frac{\partial^{2}\varphi }{\partial x\partial t}\right)\frac{\;T_{1}}{z_{1}^{}D_{1}}+\frac{\partial c_{1}}{\partial x}\right)\right]\left(1,t\right).$$

Equation (9) is obtained from the continuity condition for counterion flux, *j*_1_, at the solution/membrane interface [35,36]:

(10) $$j_{1}(1,t)=-D_{1}\left(\frac{\partial c_{1}}{\partial x}+z_{1}c_{1}\frac{\partial \varphi }{\partial x}\right)\left(1,t\right)=\frac{T_{1}}{z_{1}}\left(i+\varepsilon \frac{\partial^{2}\varphi }{\partial x\partial t}\right),$$

where *T*_1_ is the effective counterion transport number in the membrane, which is the fraction of the conduction current density carried by this kind of ions [35,36]: *T*_1_ = *j*_1_*z*_1_*/i*_c_. Since the conduction current is transferred by the ions of both kinds, *j*_1_ and *j*_2_, *T*_1_ + *T*_2_ = 1. In an ion-exchange membrane immersed in a dilute electrolyte solution, counterions are nearly the only carriers of the current, hence, *T*_1_ is close to 1 [23].

The second variant applies Equation (11):

(11) $$\left(\frac{\partial \varphi }{\partial x}\right)\left(1,t\right)=-\left(\frac{i+\varepsilon \frac{\partial^{2}\varphi }{\partial x\partial t}+z_{1}D_{1}\frac{\partial c_{1}}{\partial x}+z_{2}D_{2}\frac{\partial c_{2}}{\partial x}}{z_{1}^{2}D_{1}c_{1}+z_{2}^{2}D_{2}c_{2}}\right)\left(1,t\right)$$

which is obtained from the equation for the total current density (4) [27] written at *x =* 1:

(12) $$i(1,t)=\sum_{i=1}^{2}z_{i}\left(-z_{i}D_{i}c_{i}\frac{\partial \varphi }{\partial x}-D_{i}\frac{\partial c_{i}}{\partial x}\right)(1,t)-\varepsilon \frac{\partial^{2}\varphi }{\partial x\partial t}(1,t).$$

Note that similar approach where the total current density is used for setting boundary condition was applied by Manzanares et al. [27]. However, instead of the potential gradient expressed as a function of *i* at the solution/membrane boundary, Equations (9) or (11), the authors of Reference [27] used the mixed derivative of the electric potential:

(13) $$-\varepsilon \left(\frac{\partial^{2}\varphi }{\partial x\partial t}\right)\left(0,t\right)=i(t)+D\left[\left(\frac{\partial c_{1}}{\partial x}+c_{1}\frac{\partial \varphi }{\partial x}\right)-\left(\frac{\partial c_{2}}{\partial x}-c_{2}\frac{\partial \varphi }{\partial x}\right)\right]\left(0,t\right),$$

where *D*_1_ = *D*_2_ = *D*, *z*_1_ = 1, *z*_2_ = −1.

Computational cost of the calculation made using condition expressed by Equation (11) is lower than that when using Condition (13) (see Appendix A). 

One more condition is required at *x =* 1 to solve the equation for the co-ion concentration (*c*_2_). In this study, the following equation describing the continuity of the co-ion flux at the membrane/solution boundary is used:

(14) $$\left(\frac{\partial c_{2}}{\partial x}+z_{2}c_{2}\frac{\partial \varphi }{\partial x}\right)\left(1,t\right)=\frac{\left(1-T_{1}\right)}{z_{2}D_{2}}i.$$

Thus, Condition (8) is used to solve the equation for *c*_1_(*x*, *t*), Condition (9) or (11), to solve the equation for φ(x,t), and Condition (14), to solve the equation for *c*_2_(*x*, *t*).

The potential drop in the system is determined from the numerical solution of the boundary value problem (1–3), (6–9), (11), and (14) as:

(15) Δφ(t)=φ(1,t)−φ(0,t)=φ(1,t).

#### 2.1.2. Numerical Solution

The numerical solution of the problem formulated above was obtained by the finite element discretization using the commercially available COMSOL software package (see Appendix B).

#### 2.1.3. Parameters Used in Computations

When choosing the values of parameters, we had in mind the conditions of a chronopotentiometric experiment [37], where an electrodialysis cell with cation- and anion-exchange membranes was used. The membrane active area was 2 × 2 cm^2^, the intermembrane distance *H* = 6.5× 10^−3^ m, the temperature *T* = 293 K. A 20 mM (20 mol/m^3^) NaCl solution was flowing between the membranes with an average velocity of *V*_0_ = 4 × 10^−3^ m/c. These parameters allow calculating the thickness of the diffusion layer *δ* = 2.44 × 10^−4^ m by using the Leveque equation presented above. The estimation of the Debye length gives *L_D_* = 9.7 × 10^−8^ m, which yields *ε* = 1.6 × 10^−10^. The most computations are made at *ε* = 3 × 10^−7^, which corresponds to *L_D_* = 2.17 × 10^−9^ m relating to *c*_0_ = 0.01 mol/m^3^ subjected that *δ* remains the same as above. The increased value of *ε* is taken because of computational complexity, which grows with decreasing *ε*. The other parameters were taken from Reference [37]: The diffusion coefficients of cations *D*_1_ = 1.18 × 10^−9^ m^2^/s and anions *D*_2_ = 1.80 × 10^−9^ m^2^/s, respectively; the NaCl diffusion coefficient *D* = 1.43 × 10^−9^ m^2^/s; the cation transport number in the membrane *T*_1_ = 0.972 and that in the solution *t*_1_ = 0.395; the ion charge numbers *z*_1_ = 1, *z*_2_ = −1. To simplify the numerical solution, the ratio of the counterion concentration at the solution/CEM boundary to its value in the bulk solution *N*_c_ was taken as *N*_c_ = 1. This value is less than in real systems [2], however, as Urtenov et al. [38] have shown, when *N*_c_ ≥ 1, the value *N*_c_ does not essentially affect the distribution of concentrations and potential in the extended SCR. In the most of the computations, the current density *i/i*_lim_ = 2, where *i*_lim_ = 1/(*T*_1_ − *t*_1_) is the dimensionless limiting current density, found through the corresponding dimensional quantity, ${\ddot{i}}_{\lim}$, by using the Peers equation [35]:

(16) $${\ddot{i}}_{\lim}=Dc_{0}F/(\delta (T1-t1)).$$

#### 2.1.4. Choice of the Boundary Conditions to Set the Current Density

To estimate the impact of the boundary conditions used to specify the constant current density, two computations for system (1–3), (6–8), and (14) were performed for the case *ε* = 3 × 10^−7^, *i/i*_lim_ = 2, one applying Equation (9) and the other applying Equation (11).

The error of the calculations was estimated by the maximum difference in the values of the given current density *i* and the current density $\tilde{i}$, calculated at the point *x* = 1 using Formula (12) over the time interval from 0 to *t’*:

(17) $$\gamma =\frac{\left|\underset{\left[0,t’\right]}{\max}\left(i-\tilde{i}\left(1,t\right)\right)\right|}{i}100\%.$$

The accuracy of calculations increases with increasing *ε*. Indeed, the thickness of the quasi-equilibrium EDL, where high gradients of concentration and potential occur, is of the order of $\sqrt{\varepsilon }$. To obtain a correct numerical solution, the discretization step size should be at least $\sqrt{\varepsilon }/10$. Note that in our computations, the step size was less within the EDL and it decreased when approaching *x* = 1. In the case of *ε* = 3 × 10^−7^ and *i/i*_lim_ = 2, time *t’ =* 0.24 was chosen as related to a state, where concentration polarization is quite developed, the potential drop is close to 65 (1.6 V), while the system is still non-stationary.

**Figure A1:**
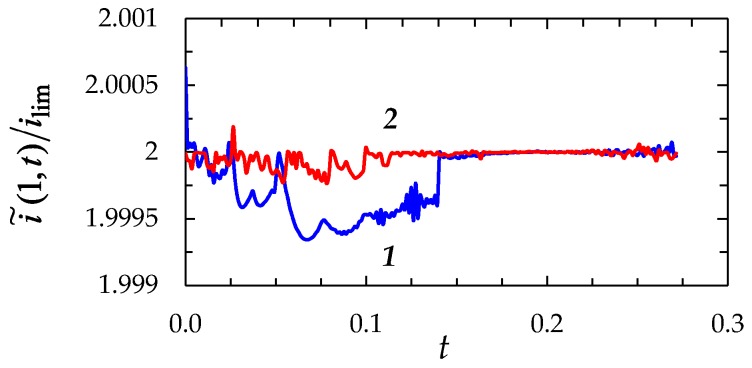
Dependence of the dimensionless current density on time $\tilde{i}(1,t)/i_{\lim}$ obtained from the numerical solution of problem (1–3), (6–8), (14) (the “primary” model) and then applying Formula (12) for the case *ε* = 3 × 10^−7^, *i*/*i*_lim_ = 2. Curves 1 and 2 are calculated using Conditions (9) and (11), respectively.

The results of computation of $\tilde{i}\left(1,t\right)$ as a function of time, performed using Conditions (9) or (11), are shown in Figure A1 in Appendix C. The errors of calculation with both conditions are small, but the use of Condition (11) allows achieving less errors, <0.016% according to Equation (17), i.e., two times less than in the case of Condition (9). Therefore, in the subsequent calculations boundary Condition (11) was used.

#### 2.1.5. Results

**Figure 2:**
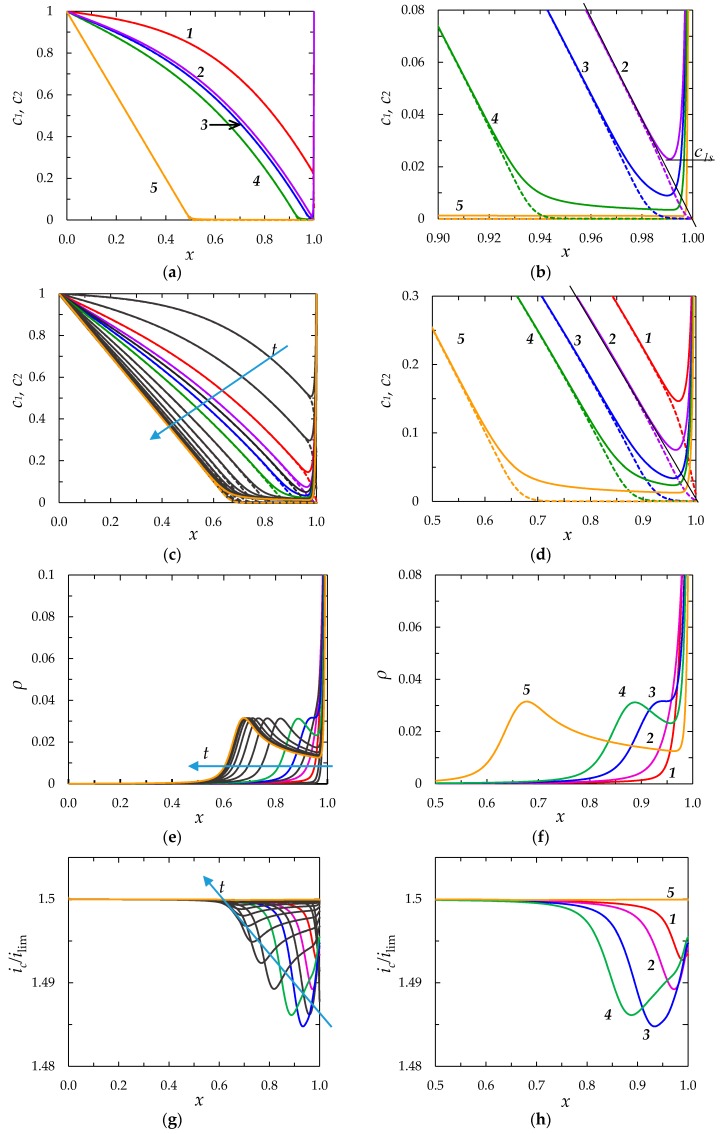
(**a**,**c**) Concentration profiles of cation (*c*_1_, the solid lines) and anion (*c*_2_, the dashed lines); (**b**,**d**) enlarged fragment of (**a**,**c**), respectively; (**e**) distribution of the dimensionless space charge density, *ρ = z_1_c_1_ + z_2_c_2_*; (**f**) enlarged fragment of (**e**); (**g**) distribution of the dimensionless conduction current density, *i*_c_*/i*_lim_; (**h**) enlarged fragment of (**g**). (**a**,**b**) show numerical calculations at *t =* 0.119 (1), 0.197 (2), 0.208 (3), 0.239 (4) and 0.621 (5) for *ε* = 3 × 10^−7^, *i/i_lim_* = 2. (**c**,**e**,**h**) show numerical calculations at *t* = 0.1, 0.2, … 1.5; (**d**,**f**,**h**), at *t =* 0.3 (1), 0.368 (2), 0.442 (3), 0.5 (4) and 1.5 (5) for *ε* = 3 × 10^−5^, *i/i_lim_* = 1.5. The other parameters are given in Section 2.1.3.

Let us consider the changes occurring in the diffusion layer with time under the action of an applied direct current (DC). Figure 2 shows the concentration profiles, as well as the distribution of the space charge density, conduction current density at different times elapsed after the switching on the current.

##### Concentration Profiles, Distribution of Space Charge and Current Density

As follows from Figure 2, the concentrations of cations and anions near the membrane surface decreases over time (Figure 2a–d). When the tangent to the electrolyte concentration profile approaches zero at *x* = 1 (curves 2), the extended SCR is starting to form at the outer edge of the quasi-equilibrium EDL (Figure 2d). A local maximum on the graph of the space charge density is formed at *t* = 0.442 (Figure 2e,f, curve 3). This maximum moves with time from the point on the outer edge of the quasi-equilibrium EDL, where the earliest maximum is formed (curve 3 in Figure 2f), towards the bulk. The minimum value of the counterion concentration, *c*_1s_, continues to decrease with time after the extended SCR formation (Figure 2b).

*Definition of transition time.* An important characteristic of transition behavior of electrode and membrane systems under an applied DC current is the transition time, *τ*. *τ* determines the time required to the near-surface concentration of electrolyte to achieve a value much less than the bulk concentration (*c*_1s_ << *c*_0_). When such a critical low concentration is attained, additional transfer mechanisms arise: In addition to electrodiffusion ion transfer, gravitational convection and/or electroconvection appear [25]; generation of additional current carriers, such as the H^+^ and OH^−^ ions in membrane systems, can occur at the interface [26]. Occurrence of additional transfer mechanisms slows down the growth of potential drop, which allows system to reach a stationary state.

First, a theory allowing calculation of *τ* was developed by Sand [39]. The theory considered an infinite stagnant diffusion layer and applied the LEN assumption. The transition time was defined as related to the state where the electrolyte concentration at the surface reached zero. The Sand transition time, *τ_S_*, is given by the following equation (adapted to the membrane systems by Krol et al. [19]):

(18) $$\tau_{S}=\frac{\pi }{4}\frac{z_{1}^{2}}{{\left(T_{1}-t_{1}\right)}^{2}}\frac{1}{i_{}^{2}}.$$

In our model, at *ε* ≠ 0, the minimum counterion concentration near the interface, *c*_1s_, never reaches zero at any current density. Hence, the transition time can be defined as the time *τ*_m_, at which the tangent to the electrolyte concentration profile crosses zero at *x* = 1 (in Figure 2b, *τ*_m_ relates to *t* = 0.197; in Figure 2d, to *t* = 0.368). When *ε* → 0, *c*_1s_ → 0.

In Figure 2, curves 1 relate to *t* < *τ*_m_; curves 2, to *t* = *τ*_m_; curves 3, to the moment *t* > *τ*_m_, where a maximum appears on the *ρ*(*x*) curve, and one can talk about an extended SCR; curves 4 relate to a state, where the extended SCR is well developed; and curves 5 to the steady state.

*Distribution of current density*. The distribution of the conduction current density over *x* at different times is shown in Figure 2g,h. According to Equation (5), when *ρ* increases with time within an interval of *x*, *i*_c_ decreases with increasing *x* inside this interval. This situation occurs within the EDL at *t* < *τ*_m_. A part of cations moving from the diffusion layer to the membrane is consumed for charging the EDL; that causes an increase of *i*_c_ as far as the cations approach the membrane (Figure 2h). Note that within the SCR, the concentration of co-ions is negligible, hence, the behavior of *ρ*(*x*) can be analyzed by the behavior of counterion concentration, *c*_1_(*x*). While *c*_1_ increases with time in a vicinity of the outer edge of the quasi-equilibrium EDL (close to *x* = 0.95 in Figure 2f), *c*_1_ goes down in the left vicinity of *x* = 1.0. The decrease in *ρ* in this vicinity causes there an increase in *i*_c_. When the maximum on the *ρ*(*x*) curve shifts from the membrane surface toward the bulk solution, *i*_c_(*x*) passes through a minimum in the point *x* corresponding to the maximum of *ρ*(*x*).

##### Chronopotentiograms

**Figure 3:**
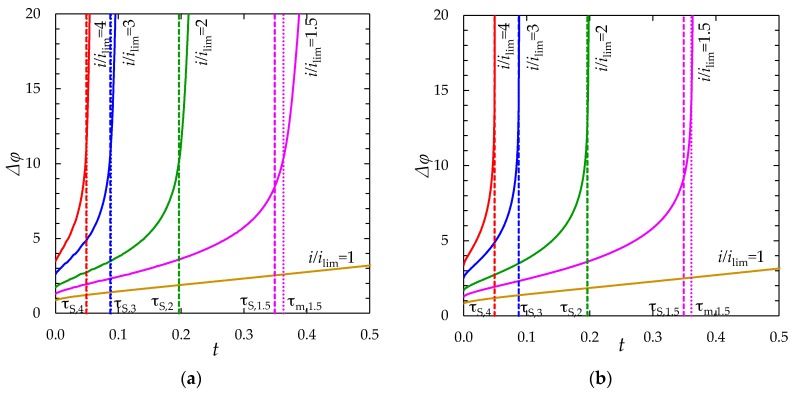
Chronopotentiograms (solid lines) computed for a CEM in a NaCl solution at *ε =* 3 × 10^−7^ (**a**) and *ε* = 1.6 × 10^−10^ (**b**) at the different current densities *i/i*_lim_ = 1; 1.5; 2; 3; 4. The dashed lines show the transition times, *τ_S_*, calculated using the Sand equation, Equation (18), at the current density indicated near the curve: *τ_S_*_,1.5_ = 0.349, *τ*_S,2_ = 0.196, *τ*_S,3_ = 0.087, *τ*_S,4_ = 0.049. The dotted lines show the transition times *τ*_m,1.5_ = 0.363 at *ε* = 3 × 10^−7^ and *τ*_m,1.5_ = 0.361 at *ε* = 1.6 × 10^−10^; the difference between *τ*_m_ and *τ*_S_ becomes negligible at *i/i*_lim_ ≥ 2. Curves (**a**) are computed using the “primary” model, curves (**b**), using the “simplified” model.

Figure 3 shows the chronopotentiograms (the dependence of the potential drop across the membrane on the time in conditions where a DC current is applied) calculated for *i/i*_lim_ = 1; 1.5; 2; 3 and 4. The dashed straight lines show the transition time, estimated using the Sand Equation (18). The dotted lines show the model transition times, *τ*_m_.

In experiment, current-induced additional mechanisms of ion transfer cause a decrease in the rate of the near-surface ion concentration decline with time [19,40]. As a result, the growth of the potential drop slows down until the system reaches a stationary state. The slowing down of the rate of the electric potential growth leads to the appearance of an inflection point on the chronopotentiometric curve, which is usually used for experimental determination of the transition time [37]. One of the possible mechanisms of slowing down the electric potential growth is electroconvection, especially that developing as electroosmosis of the second kind [41,42,43], which starts when the extended SCR is formed. The model under consideration does not consider these additional transfer mechanisms, therefore the inflection point on the calculated chronopotentiometric curve is not observed. The theoretical curve Δφ(t) continues to rise steeply, even when the extended SCR is formed. However, Δφ does tend to infinity with increasing time, which occurs when the LEN assumption is used instead of the Poisson equation. In the NPP model, at *t* → ∞, Δφ approaches a finite value, which is a very great though. Thus, at *ε* = 3 × 10^−7^, *i/i*_lim_ = 2, the steady-state value of potential drop, $\Delta \varphi_{st}\approx 30V$. In real membrane systems, $\Delta \varphi_{st}$ is of the order of 1 V [25,26], which is mainly due to the development of current-induced convection, as mentioned above.

The application of Equation (18) in the considered case of *ε* = 3 × 10^−7^, *i/i*_lim_ = 2 gives $\tau_{S}$ = 0.196 (when using our dimensionless parameters). Even when *t* only slightly exceeds *τ*_m_, there is a very large increase in Δφ(t). The computations show that *τ*_m_ (defined above, see also Figure 2b) is equal to 0.197, hence, very close to $\tau_{S}$. However, when *i/i*_lim_ < 2, *τ*_m_ is noticeably higher than $\tau_{S}$ (Figure 3). This discrepancy between our model and the Sand theory is mainly explained by the fact that we consider a diffusion boundary layer of a finite thickness *δ*, while in the Sand theory the diffusion layer is infinitely large. As it was mentioned above, Equation (18) was derived for an infinitely large stagnant layer, at the outer boundary of which the diffusion flux was zero. However, when *δ* is finite, the concentration gradient in solution and, hence, the rate of diffusion, which partially compensates the decrease in concentration caused by the current flow, is higher than when assuming *δ =* ∞; at the outer edge of the diffusion layer where the electrolyte concentration is equal to its value in the bulk solution, the concentration gradient is not zero (Figure 2a, curve 1). Therefore, the time, needed to *c*_1s_ to attain the critical value sufficient to the onset of current-induced additional transport mechanisms, is higher in the case of finite *δ* [26]. However, with increasing *i/i*_lim_ ratio, the thickness of the near-membrane region, in which concentration changes occur, decreases [26,44]. Correspondingly, the Sand’s boundary condition about zero concentration gradient at the outer edge of the diffusion layer is fulfilled better. In the conditions of the experiment described above, the difference between *τ*_m_ and $\tau_{S}$ becomes negligible at *i/i*_lim_ ≥ 2 [26,44]. With decreasing *ε*, curve Δφ(t) goes up steeper and *τ*_m_ decreases at the same *i/i*_lim_ ratio (Figure 3).

As was mentioned above, the accuracy of computation decreases with decreasing *ε*. Our computations for solving Equations (1–3), (6–8), (11) and (14) constituting the “primary” model were limited by *ε =* 3 × 10^−7^ when the results were acceptable. The accuracy of calculation of functions *c*_1_(*x*,*t*), *c*_2_(*x*,*t*), *φ*(*x*,*t*) and *ρ*(*x*,*t*) was sufficiently good: The values of the functions in the quasi-steady state calculated by the proposed galvanostatic and known potentiostatic [2] models for identical parameters differ by less than 0.1%. However, the computation of their derivatives was not always accurate, in particular, *i*(*x*,*t*) was calculated with an error larger than that in the case of the functions listed above: The maximum calculation error of the current density in the quasi-steady state in comparison with the given current density was approximately 0.5%. Nevertheless, at *ε =* 3 × 10^−5^ all the functions and their derivatives (including *i*(*x*,*t*)) were calculated with a good accuracy: The relative error is less than 0.1%.

### 2.2. The “Zonal” Model

#### 2.2.1. Decomposition of the Problem

The described above model is a complex computational problem, since it suggests a numerical solution simultaneously in a macroscopic (a few hundreds of µm) and in a microscopic (of the order of ten nanometers) regions. To obtain a sufficient accuracy, in the case of computing the curves shown in Figure 3a, it was necessary to have 75,000 elements in the computational mesh. The computational complexity of the problem can be reduced, if the region under consideration 0≤x≤1 is divided into two zones: Zone I ($0\leq x\leq 1-\delta_{3}$), which includes the electroneutral region *δ*_1_ and the extended SCR *δ*_2_; and zone II ($1-\delta_{3}\leq x\leq 1$), which is the region of equilibrium EDL *δ*_3_ (Figure 1).

As shown by Urtenov et al. [38], the steady-state solution of the NPP equations in zone I is almost independent of the counterion concentration on the right-hand boundary of the system (parameter *N*_c_ in Equation (8)). The curves *c*_1_(*x*) and *c*_2_(*x*) do not depend on the value of *N*_c_ > 1 in the interval from *x* = 0 to $x=1-\delta_{3}$; *c*_1_ reaches its minimum at $x=1-\delta_{3}$ (Figure 3). The curves *c*_1_(*x*) and *c*_2_(*x*), independent of *N*_c_, in the region $0\leq x\leq 1-\delta_{3}$ can be calculated numerically from the solution of the NPP equations using the boundary condition,

(19) $$\frac{\partial c_{1}}{\partial x}(1-\delta_{3},t)=0,$$

instead of Condition (8).

The coordinate of the point ($1-\delta_{3}$), which determines the right boundary of zone I, can be found using the formula for the thickness of the quasi-equilibrium region of the EDL *δ*_3_ [30]:

(20) $$\delta_{3}=\sqrt{\frac{2\varepsilon }{c_{1s}}}-\sqrt{\frac{2\varepsilon }{c_{1m}}},$$

where $c_{1s}=c_{1}(1-\delta_{3})$, *c*_1m_ = *c*_1_(1). In real systems, *c*_1m_ has the same order of magnitude as the fixed ions concentration in the membrane (10^3^ mol/m^3^), which is usually at least one order of magnitude higher than the bulk concentration. Meanwhile, *c*_1s_ is of the order of $\sqrt{\varepsilon }$ [38]. Therefore, the second term in Equation (18) can be neglected, when *ε* ≤ 10^−4^.

The potential drop in zone ($0\leq x\leq 1-\delta_{3}$) is found from the numerical solution in zone I:

(21) $$\Delta \varphi_{I}=\varphi \left(1-\delta_{3}\right)-\varphi \left(0\right)=\varphi \left(1-\delta_{3}\right).$$

In the stationary state, the approximate solution of the NPP equations in the second zone ($1-\delta_{3}\leq x\leq 1$) can be described by analytical Formulas (22–24) [30]:

(22) $$c_{1}=\frac{\varepsilon }{2}{\left[\frac{1}{E_{m}}+\frac{1}{2}\left(1-x\right)\right]}^{-2},$$

(23) $$c_{2}<<c_{1},$$

(24) $$\varphi =\varphi \left(1-\delta_{3}\right)+\ln\left(\frac{c_{1s}}{2\varepsilon }{\left(\frac{2}{E_{m}}+1-x\right)}^{2}\right),$$

where $E_{m}=\sqrt{2c_{1m}/\varepsilon }$ is the dimensionless electric field strength at *x =* 1.

The potential drop in the equilibrium EDL ($1-\delta_{3}\leq x\leq 1$) is determined from Equation (24) taking into account (20):

(25) $$\Delta \varphi_{II}\left(t\right)=-\ln\frac{c_{1m}}{c_{1s}}=-\ln\frac{N_{c}}{c_{1}\left(1-\delta_{3},t\right)}.$$

The total potential drop in the system (0≤x≤1) is the sum of the potential drops in the first and the second zones:

(26) $$\Delta \varphi \left(t\right)=\Delta \varphi_{I}\left(t\right)+\Delta \varphi_{II}\left(t\right).$$

Note that Equation (25) can also be obtained from the Nernst–Planck equation written for counterion, if it is assumed that the terms corresponding to the contribution of diffusion and electromigration are much larger than their difference, *j*_1_. Then we obtain the Boltzmann distribution of concentrations, the integration of which gives Equation (25). Such distribution can take place, if the EDL remains quasi-equilibrium despite the flow of electric current and the non-stationary state of the system.

In conditions of a DC current applied, the time of establishing a stationary concentration profile in a quasi-equilibrium EDL is of the order of $L_{D}^{2}/D$ [45], where $L_{D}=\sqrt{RT\varepsilon_{0}\varepsilon_{r}/\left(2c_{0}z_{1}^{2}F^{2}\right)}$ is the Debye length. This time is about 10^–5^ s, if the Debye length is estimated as 100 nm and the diffusion coefficient as 10^–9^ m^2^/s. The corresponding time for the diffusion layer is *δ*^2^/*D* = 10 s (at *δ* ~ 100 µm) [45]. Thus, the state of the equilibrium part of the EDL can be considered as quasi-stationary: When the *c*_1s_ concentration changes, the profiles of concentration and potential in the EDL, which depend on *c*_1s_, are established almost instantaneously. This circumstance significantly facilitates the solution of non-stationary problems using the NPP equations (excepting the cases where the current density varies with a period comparable with the time of 10^–5^ s or less, characteristic for the charging time of the EDL). The distributions of concentrations and potential are searched separately in zones I and II, and then the obtained solutions are “stitched” by using the condition of equality of concentration *c*_1s_ in the both zones. In zone I, a non-stationary solution is found numerically using Equations (1–3), (7), (11), (14), (15) and boundary Condition (19) on the right-hand boundary. In zone II, Equations (20–26) are used as the solution, where the concentration *c*_1s_ at the left-hand boundary is determined from the numerical solution in zone I. It is important that a simple stationary solution for zone II may be applied for solving a complex non-stationary problem set for the region comprising zones I and II.

Our approach where numerical solution in one part of the system is complemented with an analytical solution in the other part (quasi-equilibrium EDL), is similar to the approach of Rubinstein and Zaltzman [3,28,46]. The difference is that in the cited works, a numerical solution is found for the electroneutral region; in the SCR, which consists of the extended SCR and the quasi-equilibrium EDL, an approximate analytical solution taking into account the electroosmotic slip of the solution is used. In our case the zone where the analytical solution is applied is thinner than that in the approach [3], hence why the analytical solution may be obtained simpler and more precise. In this paper, we consider the 1D case for the sake of simplicity needed to better understand the advantages, which can be obtained when applying this approach. The approach can be further extended to 2D and eventually to 3D systems, due to the simplicity of the analytical solution in the quasi-equilibrium EDL. 

#### 2.2.2. Comparison of the “Primary” and “Zonal” Models

**Figure 4:**
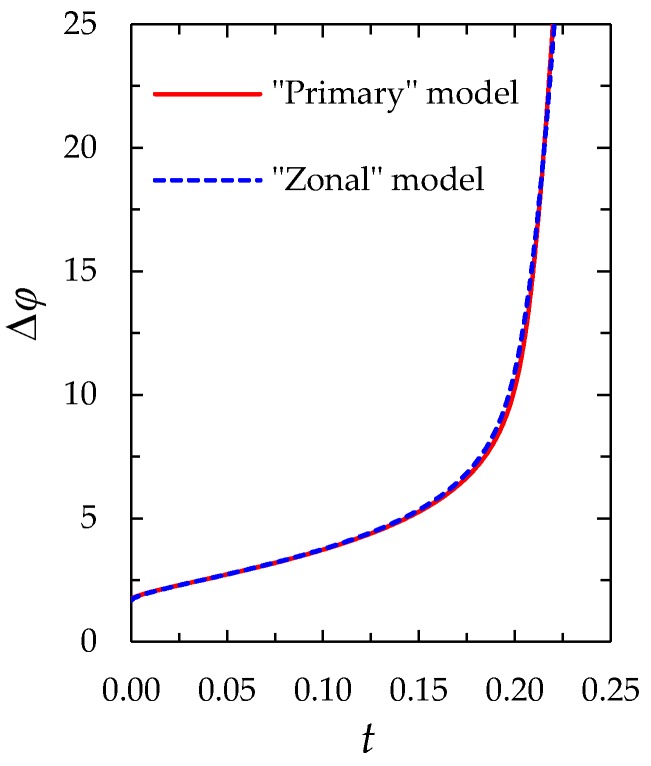
Chronopotentiograms of a CEM at *i/i*_lim_ = 2, *ε =* 3 × 10^−7^. The solid line is calculated using the “primary” model; the dashed line, using the “zonal” model.

**Figure 5:**
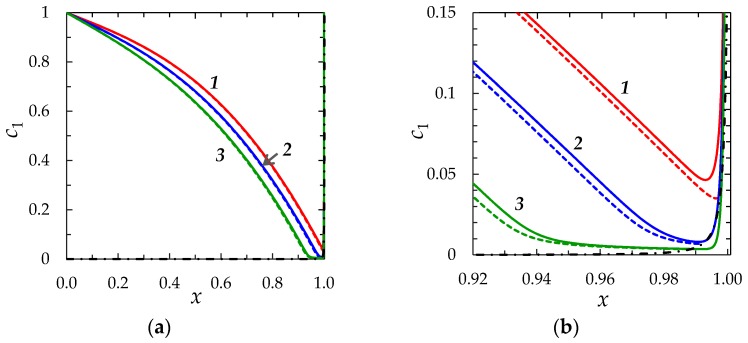
(**a**) Concentration profiles of cations near a CEM for *ε =* 3 × 10^−7^ and *i /i*_lim_ = 2 at three different instants of time: *t* = 0.189 (1), 0.213 (2) and 0.240 (3). The solid lines are calculated numerically using the “primary” model (the region of applicability 0 ≤ *x* ≤ 1), the dashed lines are calculated numerically using the “zonal” model in the region 0 ≤ *x* ≤ 1 − *δ*_3_, the dash-dotted line is the result of calculation of *c*_1_ by Formula (22) (the region of applicability is 1 − *δ*_3_ ≤ *x* ≤ 1); (**b**) enlarged fragment of (**a**).

The results computed using the both models are compared for the same parameters (given in Section 2.1.3). Figure 4 and Figure 5 show the chronopotentiograms and the concentration profiles, respectively.

**Figure 6:**
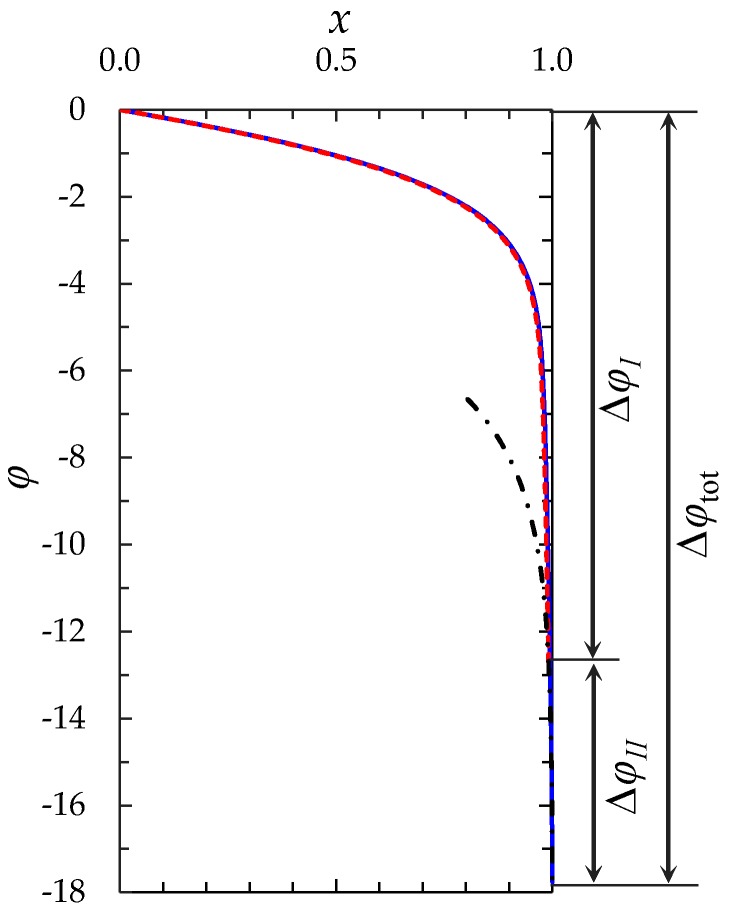
Distribution of the electric potential for *i/i*_lim_ = 2 at time t = 0.213, *ε =* 3 × 10^−7^. The solid line is calculated numerically using the “primary” model (the region of applicability is 0 ≤ *x* ≤ 1), the dashed line is calculated using the “zonal” model (the region of applicability is 0 ≤ *x* ≤ 1 − *δ*_3_), the dash-dotted line is the result of calculation of φ by Formula (24) (the region of applicability is 1 − *δ*_3_ ≤ *x* ≤ 1).

Figure 6 shows the results of calculation of the electric potential distribution at *t* = 0.213 using the “primary” and “zonal” models.

It can be seen from Figure 4, Figure 5 and Figure 6, that the results of calculation using the “zonal” and “primary” models are in a quite good quantitative agreement (the values of the concentrations and potential differ by less than 1% everywhere, except in the regions where they are characterized by a large gradient; in these regions the difference increases to 20%). As for the transition time, in the case of *ε =* 3 × 10^−7^, at *i/i*_lim_ = 1.5; 2; 3, the zonal model gives *τ*_m_ = 0.360, 0.195 and 0.087, while the “primary” model yields *τ*_m_ = 0.363, 0.197 and 0.088, respectively. The main reason of deviation is due to the fact that the *c*_1_(*x*) and φ(x) functions according to Equations (22) and (24) do not depend on time and the stage of concentration polarization within the quasi-equilibrium EDL. However, this dependence, although weak, exists and is taken into account in the “primary” model.

As shown in Appendix D, the use of “zonal” model allows reducing the number of mesh elements 75 times and the total calculation time, 35 times, in comparison with the “primary” model, when calculating chronopotentiograms for the case of *ε =* 3 × 10^−7^, *i/i*_lim_ = 2.

### 2.3. The “Simplified” Model

#### Idea and Limits of Applicability of the “Simplified” Model

The computational time needed when using the “zonal” model is increased by the calculations related to the necessity to take into account the change of the first zone thickness (1 − *δ*_3_) with time, Equation (20). In addition, this complicates the algorithm of the problem solution. In the Comsol Multiphysics environment this requires the use of the special module that implements a change in geometry. The “zonal” model can be simplified if the thickness of the equilibrium part of the SCR is neglected in comparison with the thickness of the diffusion layer, i.e., *δ*_3_ = 0 is assumed. That is, Condition (19) on the right-hand boundary of zone I is moved to the point *x* = 1. Such simplification does not mean that we neglect the potential drop $\Delta \varphi_{II}$ in zone II. The value of $\Delta \varphi_{II}$ is calculated using Equation (25), where the value of *c*_1s_ is found from the numerical solution as a value of *c*_1_ at the point *x* = 1.

It can be said that the solution is sought for the part of the diffusion layer (including the electroneutral zone and the extended SCR), whose thickness is not (1 − *δ*_3_) (as in the reality), but is assumed to be 1.

Our estimates show that the computational time needed to solve the «simplified» model is approximately 38% less compared to the time of the similar calculations with the “zonal” model (Appendix D, Table A2).

**Figure 7:**
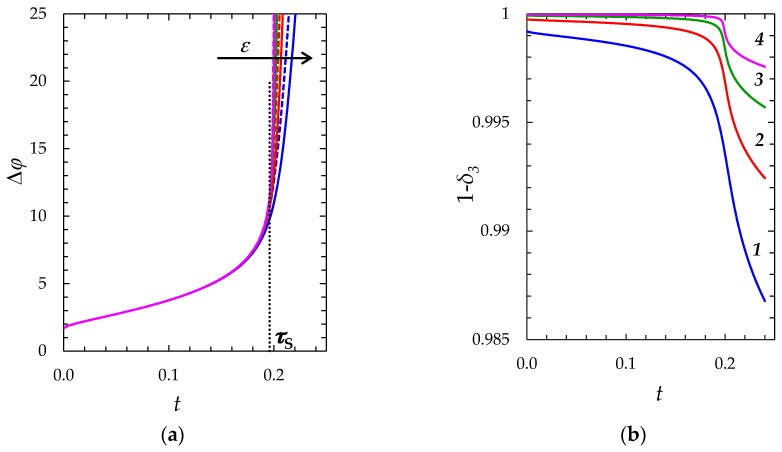
(**a**) Chronopotentiograms calculated using the “zonal” model (solid lines) and the “simplified” model (dashed lines), *τ*_S_ = 0.196 is the transition time determined using the Sand formula, Equation (18); (**b**) time dependence of the dimensionless thickness of the first zone (1 *− δ_3_*) found using the “zonal” model. The calculations are carried out at *i*/*i*_lim_ = 2 for the following values of *ε*: 3 × 10^−7^ (1); 3 × 10^−8^ (2); 3 × 10^−9^ (*3*); 3 × 10^−10^ (4).

To determine conditions under which the reduction of the first zone thickness by the value of *δ*_3_ can be neglected, we performed a series of computational experiments using the “zonal” model taking into account the nonzero thickness of the equilibrium part of the SCR, Formula (18), and when assuming it zero (*δ*_3_ = 0, “simplified” model) for the fixed dimensional thickness of the diffusion layer 244.32 µm and different *ε*: 3 × 10^−7^ (*c*_0_ = 0.01 mol/m^3^); 3 × 10^−8^ (*c*_0_ = 0.1 mol/m^3^); 3 × 10^−9^ (*c*_0_ = 1 mol/m^3^); 3 × 10^−10^ (*c*_0_ = 10 mol/m^3^). The calculated chronopotentiograms are shown in Figure 7a,b. It can be seen, that with decreasing *ε* (increasing the bulk concentration *c*_0_), the slope of the chronopotentiograms at *t* > *τ*_S_ increases. Therefore, to estimate the influence of *ε* on the accuracy of the “simplified” model, the errors are determined at the time $\tilde{t}$, which relates to the total potential drop $\Delta \varphi \left(\tilde{t}\right)=20$ (see Table 1).

The time at which Δφ=20, found using the “primary” model in the case of *ε* = 3 × 10^−7^, agrees with the corresponding value from the “zonal” model with an accuracy of four significant digits ${\tilde{t}}_{pr}={\tilde{t}}_{zon}=0.2137$. For smaller values of *ε*, this time was not calculated using the “primary” model, due to the rapidly increasing computational complexity.

Thus, with decreasing *ε* (increasing *c*_0_), the difference in the results obtained using the “simplified” and the “zonal” models decreases. This is due to the fact that with decreasing *ε* (increasing *c*_0_), the thickness of equilibrium EDL decreases, as well as the effect of this parameter on the results of calculations. Therefore, the “simplified” model is applicable at relatively low values of *ε*, just in the conditions where the numerical solution of the problem related to the “primary” model is extremely complex.

### 2.4. Effect of Setting Condition for the Current Density at the Left-Hand Boundary

A condition setting the current density can be formulated at the left-hand boundary instead of the right-hand one, Equation (8). Then we write:

(27) $$\left(\frac{\partial \varphi }{\partial x}\right)\left(0,t\right)=-\left(\frac{i+z_{1}D_{1}\frac{\partial c_{1}}{\partial x}+z_{2}D_{2}\frac{\partial c_{2}}{\partial x}}{z_{1}^{2}D_{1}c_{1}+z_{2}^{2}D_{2}c_{2}}\right)\left(0,t\right).$$

In Equation (27) the displacement current density is omitted, since it is negligibly small at *x* = 0 (*i*_d_(0,t)/*i*_lim_ < 10^−6^).

The condition for the potential should be set at the right-hand boundary instead of the left-hand one, Equation (7):

(28) φ(1,t)=0.

**Figure 8:**
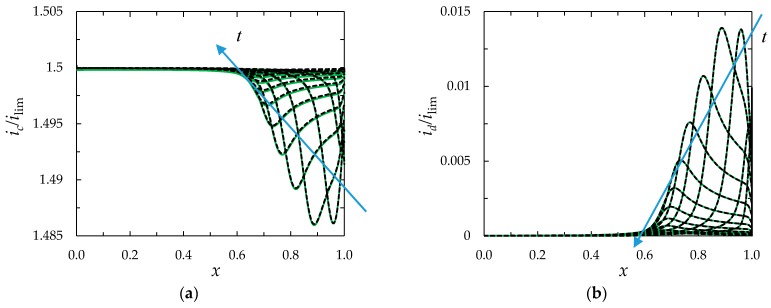
Distribution of conduction current density, *i*_c_/*i*_lim_, (**a**) and displacement current density, *i*_d_/*i*_lim_, (**b**), computed at different times, *t*, in the case of *ε* = 3 × 10^−5^, *i/i_lim_* = 1.5, *T*_1_ = 0.972, *N*_c_ = 1. The solid lines relate to the case where the current density is set at the right-hand boundary, Equations (7) and (11); the dotted lines, to the case of left-hand boundary, Equations (27) and (28); *t* = 0.1, 0.2, …, 1.5.

When Equations (27) and (28) are used, the behavior of the system is quite similar to that obtained in the case of use of Equations (11) and (7) setting the current density at the left-hand boundary and the potential at the right-hand boundary, respectively (Figure 8).

**Figure 9:**
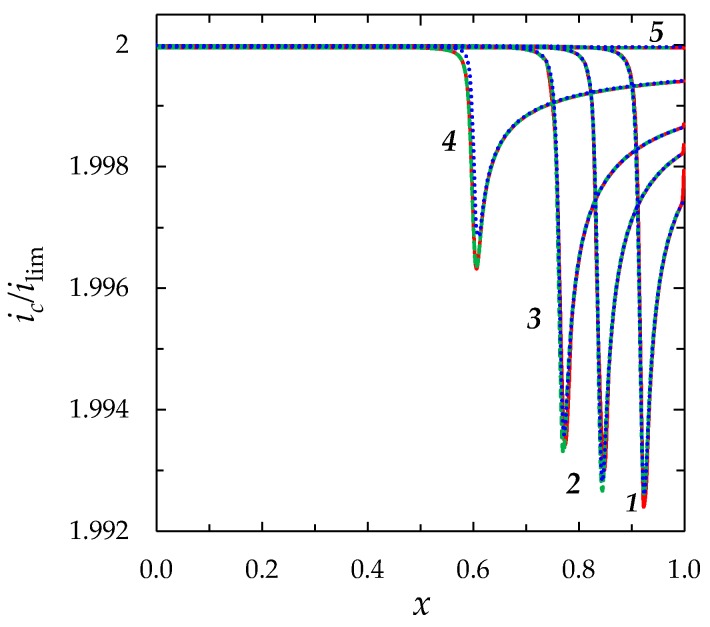
Distribution of current density *i*_c_/*i*_lim_ in the depleted diffusion layer at *t* = 0.25 (1), 0.3 (2), 0.35 (3), 0.5 (4), 1.5 (5) in the case where a constant current density is set at the left-hand boundary, Equation (27). The red solid line is computed using the “primary” model, the green dashed line, using the “zonal” model; the blue dotted line, using the “simplified” model. *ε* = 3 × 10^−7^, *i/i_lim_* = 2.

Comparison of the *i*_c_(*x*,*t*) functions computed using the “primary”, “zonal” and “simplified” models shows a very good agreement between all three models, (the values differ by less than 0.03%), Figure 9.

## 3. Comparison with the Experiment

**Figure 10:**
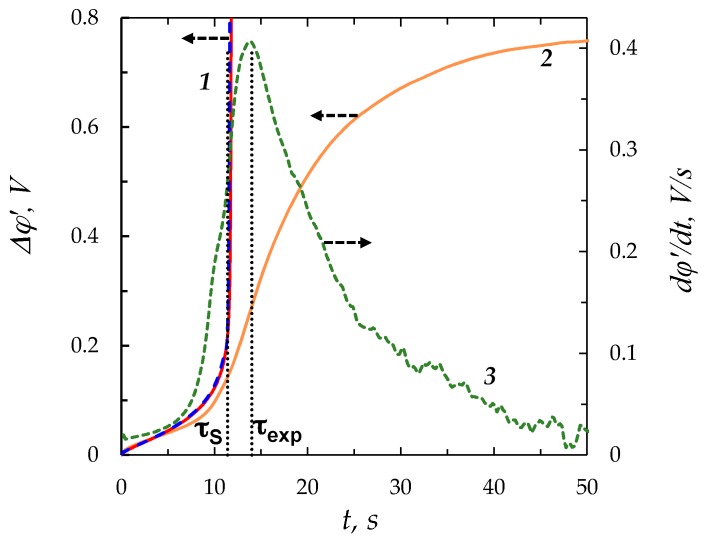
Calculated 1 (“zonal” model—solid red line, “simplified”—dashed blue line) and experimental 2 chronopotentiograms for a MK-40_MOD_ membrane in a 20 mol/m^3^ NaCl solution (*ε* = 1.6 × 10^−10^) at *i/i*_lim_ = 1.7. The graphics are plotted as the reduced potential drop ∆*φ*’ *=* ∆*φ* − ∆*φ_ohm_* vs. time (where ∆*φ_ohm_* is the ohmic potential drop in the membrane system measured just after switching on the current); the Sand (*τ_s_* = 11.4 s) and the experimental (*τ*_exp_ = 14 s) transition times are shown with vertical dotted lines; curve 3 presents the experimental values of *d*∆*φ*’*/dt*. The experimental data are taken from Reference [37].

Figure 10 shows the chronopotentiograms obtained experimentally [37] and theoretically using the “zonal” and “simplified” models. The experiment was carried out with a laboratory made cation-exchange membrane MK-40_MOD_ having electrically homogeneous surface and 0.02 M (20 mol/m^3^) NaCl solution in the conditions described in Section 2.1.3 at *i/i*_lim_ = 1.7; the corresponding value of *ε* is 1.6 × 10^−10^. In these conditions, the transition time *τ*_m_ computed using the “zonal” and “simplified” models is the same (with an accuracy of 4 significant digits) and equal to 11.53 s. The Sand transition time $\tau_{S}$, found using Equation (18) is 11.4 s. A good agreement between experimental and calculated curves is observed at times *t* < *τ*_S_ (the difference of experimental and calculated values of the potential drop is less than 10%). When *t* approaches *τ*_S_, the theoretical curve goes up steeply, while the experimental curve rises also, while not so steeply; it slows down and forms an inflection point at *τ*_exp_ ≈ 14 s, then flattens out and reaches a steady state. As we mentioned above, slowing down of the chronopotentiogram is generally due to the development of current-induced convection, which is electroconvection under the conditions of the experiment [37]. Electroconvection mixes solution near the surface: It provides an additional delivery of a more concentrated electrolyte from the solution bulk to the surface and evacuates depleted solution from the near-surface region [10,32,47,48]. The lower concentration at the surface, the more intensive is electroconvection, which results in reaching a stationary state of the experimental curve [26,37].

As Figure 10 shows, the theoretical transition time is noticeably lower than the experimental value, *τ*_exp_, which corresponds to the inflection point (the maximum of the derivative *d*∆*φ/dt*). The reason apparently is that noticeable electroconvection arises at the times lower than *τ*_S_. That leads to a delay in the concentration decreasing process at the membrane surface in comparison with the case where the ion delivery to the surface occurs only through diffusion and migration, as it is assumed in the Sand theory and in our model. As a result, the inflection point on the chronopotentiogram appears later than in the theoretical calculations. The reason of “early” electroconvection can be an electrical and geometrical inhomogeneity of the surface, which along with non-ideal selectivity of the membrane with respect to co-ion transfer can produce “equilibrium” electroconvection [29,49]. Our results shown in Figure 2 give a new insight in understanding development of the “earlier” non-stationary electroconvection. Indeed, we see that the extended SCR arises quite rapidly after reaching the transition time and the maximum of space charge density moves with time. Accordingly, current density near the surface varies in time and distance. The surface of ion-exchange membrane is not really homogeneous, so that the local current density is higher through the areas with better conductivity. As a result, the ion concentration at these areas will decrease more rapidly than at the less conductive areas [25,50]. Hence, there will be non-uniform distribution of the space charge density over the surface along the longitudinal coordinate. That produces local currents along the surface, which provide electroosmotic flow. A short-term lead or delay in the formation of SCR can generate important lateral currents.

For a more accurate description of chronopotentiograms in systems with ion-exchange membranes, the proposed one-dimensional modelling should be extended at least to two-dimensional description with addition of the Navier-Stokes equations where the action of electric force on the electric space charge in solution is taken into account.

## 4. Conclusions

Mathematical modelling of 1D non-stationary ion transfer in ion-exchange membrane systems under an applied DC current is carried out using the NPP equations. The stagnant depleted diffusion layer adjacent to the membrane is considered. A new boundary condition, expressing the potential gradient as an explicit function of current density, is proposed. Thus, a constant current density is set at the left- or right-hand boundary of the diffusion layer. The problem allows describing the concentrations of cations and anions, as well as the potential, space charge density and current density as functions of time and distance. The numerical solution of the boundary-value problem when using the new boundary condition is identical to that obtained when using the condition proposed by Manzanares et al. [27]. However, the computation time is essentially less in the case where the new condition is applied. We have found that both conditions can be set at the outer edge of the diffusion boundary layer (*x* = 0) or at the boundary between the solution and the membrane (*x* = 1): The numerical solution gives the same results. The computation time is essentially lower, when these conditions are set at *x* = 0.

It is shown that a very good approximate solution of the boundary value problem can be obtained by a combination of numerical solution in the electroneutral region and the extended SCR (zone I) and an analytical solution in the quasi-equilibrium EDL (zone II) of the depleted diffusion layer (the “zonal” model). Additional simplification may be given by neglecting the thickness of the quasi-equilibrium EDL in comparison to the diffusion layer thickness (the “simplified” model). It is shown that the “simplified” model agrees well with the “zonal” model in the range of small parameter *ε* (<10^−7^), which relates to the experimental conditions of real membrane systems. The calculation of the transition time, *τ*, for an ion-exchange membrane results in a rather good agreement with experiment. The difference between the computed and experimental values of *τ* is explained by “non-ideal” behavior of the system, mainly by the development of electroconvection, which is not considered in the actual mathematical description. The computations of the space charge density, *ρ*(*x*,*t*), suggest that rapid changes of *ρ* with time and distance could lead to lateral electroosmotic flows delaying depletion of near-surface solution.

## Figures and Tables

**Table 1:** Estimation of the deviation between the calculations of the time at which Δφ=20 when using the “zonal” and the “simplified” models for the different values of *ε.*

ε	*c*_0_, mol/m^3^	The Time at Which Δφ=20		$\mathrm{Deviation},\gamma =\frac{\left|{\tilde{t}}_{zon}-{\tilde{t}}_{simpl}\right|}{{\tilde{t}}_{zon}}100\%$
		$“\mathrm{Zonal}”\;\mathrm{Model},{\tilde{t}}_{zon}$	$“\mathrm{Simplified}”\;\mathrm{Model},\;{\tilde{t}}_{simpl}$	
$3\times 10^{-7}$	0.01	0.214	0.208	2.7
$3\times 10^{-8}$	0.1	0.204	0.202	1.3
$3\times 10^{-9}$	1	0.200	0.199	0.7
$3\times 10^{-10}$	10	0.199	0.198	0.4

## Appendix A

To compare the computational costs of calculations with using Condition (11) or Condition (13), we estimated the time (denoted by *t_st_*) required to calculate the distributions of the ion concentrations and potential when applying the “primary” model (1–3), (6–8), (11) or (13), (14) from *t* = 0 to a sufficiently long time (*t* = 1.6), at which a quasi-stationary solution is established. Calculations were performed with the same parameters (*ε* = 3 × 10^−7^, *i/i_lim_* = 2) and accuracy. Calculations using Conditions (11) or (13), where both conditions are set at the left-hand boundary, *x =* 0, or at the right-hand boundary, *x =* 1, give the identical results, but essentially differ in the required computational costs (Table A1). The calculation time, *t_st_*, when using Condition (11) is 1.3 times less than when using Condition (13), if both conditions are set at the left-hand boundary; this difference is 3.8 times, if the conditions are set at the right-hand boundary (Table A1).

All calculations were carried out by using an Intel Core i7-4930K CPU.

**Table A1:** The computation time, *t_st_*, needed when using boundary Condition (11) or (13) at the left-hand (*x =* 0) or right-hand (*x =* 1) boundary.

Boundary Condition	*t_st_*, s	
	Left-Hand Boundary	Right-Hand Boundary
Equation (11)	1866	8005
Equation (13)	2491	30,200

## Appendix B

A numerical solution was found by the finite element method using Comsol Multiphysics software package on a non-uniform computational mesh (the density of the mesh elements was increased near the solution/membrane boundary). The equations were implemented by using the following modules: “Transport of Diluted Species” for the anions and cations concentrations fields, Equations (1) and (2); “General Form PDE” for the electric potential fields, Equation (3). For time-depended calculations the algorithm of a Fully Coupled solution was used.

In the “zonal” model, to implement the change of the first zone thickness (1 − *δ*_3_) with time, the Deformed Geometry module was added to the Comsol Multiphysics modules mentioned above. The Free Deformation function of this module allows modifying the size of the geometric region of the system according to the specified boundary condition: on the right-hand boundary the Prescribed Mesh Displacement condition provides the fulfillment of Equation (20).

## Appendix D

To select the optimal computational mesh, the following convergence test was used: a calculation is carried out on some computational mesh (consisting of *K*_1_ elements) and a chronopotentiometric curve $\Delta \varphi_{K1}\left(t\right)$ is calculated for time from 0 to *t’* = 0.239 at current density *i/i*_lim_ = 2. Then the mesh is refined (*K*_2_ elements), calculations are carried out again and the value $\Delta \varphi_{K2}\left(t\right)$ is calculated. The maximal relative error of the calculation of the potential drop at the indicated time interval is determined by the formula:

(A1) $$\gamma_{1}=\underset{t\in \left[0,t’\right]}{\max}\left(\left|\Delta \varphi_{K1}\left(t\right)-\Delta \varphi_{K2}\left(t\right)\right|/\Delta \varphi_{K2}\left(t\right)\right)100\%.$$

These actions were repeated until $\gamma_{m}\leq 0.15\%,$ where *m* is the number of calculation run. The condition for calculation stopping for the “primary” model was fulfilled at *K*_m_ = 75,000 and for the “zonal” model at *K*_m_ = 1000. This is due to the fact that when using the “zonal” model, the numerical solution is searched in the region from which the zone with large gradients of concentrations and potential near the membrane surface is excluded. Thus, the application of the “zonal” model allows reducing the number of mesh elements 75 times.

The total computation time when using the “zonal” model is 35 times less than that when using the “primary” model (Table A2).

**Table A2:** The total time needed for computation of $\Delta \varphi \left(t^{\prime }\right)$ by using different models.

Model	Relative Error, *γ*	Number of Mesh Elements, *K*	The Computation Time, the Case of *t’* = 0.239, s
“Primary”	0.14%	75,000	563
“Zonal”	0.14%	1000	16
“Simplified”	0.14%	1000	10
